# Breast Carcinomatous Lymphangitis as an Unusual Presentation of Ovarian Cancer

**DOI:** 10.3390/diagnostics11112106

**Published:** 2021-11-14

**Authors:** Barbara Muoio, Giorgio Treglia, Paola Migliora, Maria Del Grande

**Affiliations:** 1 Oncology Institute of Southern Switzerland, Ente Ospedaliero Cantonale, 6501 Bellinzona, Switzerland; barbara.muoio@eoc.ch (B.M.); maria.delgrande@eoc.ch (M.D.G.); 2 Imaging Institute of Southern Switzerland, Ente Ospedaliero Cantonale, 6501 Bellinzona, Switzerland; 3 Faculty of Biomedical Sciences, Università della Svizzera italiana, 6900 Lugano, Switzerland; 4 Faculty of Biology and Medicine, University of Lausanne, 1011 Lausanne, Switzerland; 5 Cantonal Institute of Pathology, Ente Ospedaliero Cantonale, 6600 Locarno, Switzerland; paola.migliora@eoc.ch

**Keywords:** carcinomatous lymphangitis, breast, ovarian cancer, imaging, pathology

## Abstract

We describe the case of a 45-year-old woman with an unusual presentation of metastatic ovarian cancer. The patient presented to the oncological clinic with a three-week history of skin rash on the right breast. She underwent a chest and abdomen CT scan, which showed skin thickening of the right breast, right pleural effusion and bilateral cystic ovarian masses. Biopsy of a left ovarian lesion by diagnostic laparoscopy revealed the presence of ovarian serous carcinoma. Biopsy of the breast skin lesion revealed the presence of carcinomatous lymphangitis and immunohistochemistry documented the ovarian origin.

**Figure 1:**
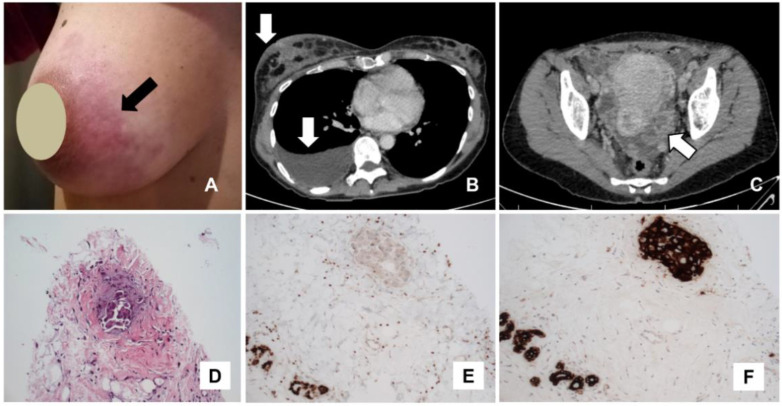
A 45-year-old woman presented to the oncological clinic with a three-week history of skin rash on the right breast ((**A**); arrow). The patient underwent a chest and abdomen CT scan (**B**,**C**), which showed skin thickening of the right breast ((**B**); upper arrow), right pleural effusion ((**B**); lower arrow) and bilateral cystic ovarian masses ((**C**); arrow). Biopsy of a left ovarian lesion by diagnostic laparoscopy revealed the presence of ovarian serous carcinoma. The patient performed other staging exams (breast ultrasound and mammography) which were suspicious for carcinomatous lymphangitis. Biopsy of the breast skin lesion revealed the presence of carcinomatous lymphangitis (**D**), whereas immunohistochemistry showed negativity for CK20, GATA3 (**E**) and positivity for CK7 (**F**), PAX8 and WT1. The immunochemistry pattern demonstrated the ovarian origin of breast lesions. After the diagnosis of metastatic disease, the patient underwent chemotherapy with carboplatin and paclitaxel with partial radiological response after three cycles. Due to inoperable disease, the patient continued chemotherapy with the addition of bevacizumab, obtaining partial treatment response at last follow-up (about one year after the diagnosis of carcinomatous lymphangitis). Carcinomatous lymphangitis may be a metastatic manifestation of different tumors; the most common primary sites are breast, lung and stomach, whereas in rare cases it can be due to ovarian cancer [1,2,3,4,5,6,7,8]. In the described case, an integrated diagnostic approach was very useful to detect breast carcinomatous lymphangitis as an uncommon presentation of metastatic ovarian cancer.

## Data Availability

Original data supporting the reported results are available contacting the authors.
